# Assessment of Ionospheric Gradient Impacts on Ground-Based Augmentation System (GBAS) Data in Guangdong Province, China

**DOI:** 10.3390/s17102313

**Published:** 2017-10-11

**Authors:** Zhipeng Wang, Shujing Wang, Yanbo Zhu, Pumin Xin

**Affiliations:** National Key Laboratory of CNS/ATM, School of Electronic and Information Engineering, Beihang University, Beijing 100191, China; wangzhipeng@buaa.edu.cn (Z.W.); wangshujing@buaa.edu.cn (S.W.); dearmin1992@buaa.edu.cn (P.X.)

**Keywords:** GBAS, ionosphere monitoring, ionospheric threat model, airworthiness assessment

## Abstract

Ionospheric delay is one of the largest and most variable sources of error for Ground-Based Augmentation System (GBAS) users because inospheric activity is unpredictable. Under normal conditions, GBAS eliminates ionospheric delays, but during extreme ionospheric storms, GBAS users and GBAS ground facilities may experience different ionospheric delays, leading to considerable differential errors and threatening the safety of users. Therefore, ionospheric monitoring and assessment are important parts of GBAS integrity monitoring. To study the effects of the ionosphere on the GBAS of Guangdong Province, China, GPS data collected from 65 reference stations were processed using the improved “Simple Truth” algorithm. In addition, the ionospheric characteristics of Guangdong Province were calculated and an ionospheric threat model was established. Finally, we evaluated the influence of the standard deviation and maximum ionospheric gradient on GBAS. The results show that, under normal ionospheric conditions, the vertical protection level of GBAS was increased by 0.8 m for the largest over bound $\sigma_{vig}$ (sigma of vertical ionospheric gradient), and in the case of the maximum ionospheric gradient conditions, the differential correction error may reach 5 m. From an airworthiness perspective, when the satellite is at a low elevation, this interference does not cause airworthiness risks, but when the satellite is at a high elevation, this interference can cause airworthiness risks.

## 1. Introduction

Ground-Based Augmentation System (GBAS) is a type of regional satellite augmentation system used for precision positioning [1]. The main function of GBAS is to support a precision approach in all-weather operations by augmenting satellite signals with real-time broadcast differential corrections and integrity information for each satellite in view [2,3]. Airborne GBAS users receive the broadcast data, which they then use to correct global navigation satellite system measurements. GBAS have extremely high accuracies and availabilities, which are necessary for Category I (CAT I) approaches, and in the future, GBAS will also support CAT II/III precision approaches [4,5]. Compared with the Instrument Landing System (ILS), GBAS have many advantages, such as greater stabilities, low signal noise, low costs, and high efficiencies [6]. At present, GBAS facilities have been installed in many airports in the United States, Britain and other countries. In the future, GBAS is expected to become one of the main take-off and landing systems for airports.

It is crucial for GBAS to provide timely warnings for users when any error or abnormality appears in the system and before the position errors exceed the confidence bounds [1]. Among the various GBAS errors, large ionospheric error is one of the most challenging [7]. This phenomenon exists because the ionosphere, which is generally stable, also produces severe and extreme ionospheric events, especially during solar activity periods [8]. During extreme ionospheric storms, GBAS users and GBAS ground facilities may experience different ionospheric delays, leading to considerable differential errors, which threaten the safety of users. To understand and reduce the impacts of the ionosphere on GBAS, the ionospheric anomaly threat model and parameter $\sigma_{vig}$ (sigma of the vertical ionospheric gradient) must be established. During the last solar maximum (2000–2004), the ionospheric anomaly threat model for the local area augmentation systems in the Conterminous United States (CONUS) was developed based on the extreme ionospheric gradients observed in the United States [9]. The CONUS model shows that the largest slant ionospheric gradient found in the United States is 413 mm/km. In Korea, an anomalous ionospheric threat model has also been established; this model shows that the maximum slant ionospheric gradient observed in South Korea region is 160 mm/km [10]. In Brazil, the largest gradient is approximately 850 mm/km, which is almost twice as large as the maximum gradient observed in the CONUS [11]. However, these models cannot be directly applied in China because ionospheric behavior varies significantly between locations with different solar radiation and geomagnetic environments. On a normal day, the residual ionospheric error between a user and GBAS can be computed using the parameter $\sigma_{vig}$, which is broadcast by the GBAS ground facility [12]. The value $\sigma_{vig}$ = 4 mm/km proposed for the CONUS is conservative [13]. To improve the GBAS performance in China, the ionosphere must be monitored and the ionospheric behavior (including $\sigma_{vig}$) must be characterized. An anomalous ionospheric threat model has been established for normal days in the Beijing area of China in our previous work [14]. However, in southern China, the latitudes are lower and the ionosphere is more active. Guangdong Province is one of the provinces in which ionospheric activity is more active; thus, this paper studies the characteristics of the ionosphere in Guangdong Province and their impact on GBAS.

In this paper, an improved “Simple Truth” algorithm, which was proposed in our previous work, was used to process the GPS data collected from 65 reference stations in Guangdong Province and to analyze the ionospheric gradient [14]. We also provide a detailed discussion of the two largest ionospheric gradient events in this paper. By simulating the ionosphere wave fronts at both aircraft and GBAS, we studied the max ionospheric gradient and its influence on GBAS, including the vertical protection level (VPL), $S_{\mathrm{vert}}$ (projection of the vertical component and the translation of the along track errors in the vertical direction, as stated in Equation (15)) and maximum differential correction error. Then, we studied the maximum differential correction error of GBAS from an airworthiness perspective.

The remainder of this paper is organized as follows: Section 2 describes the improved “Simple Truth” algorithm along with the improvements of the new algorithm. Section 3 provides a detailed examination of the two largest ionospheric gradient events, which occurred on 24–25 March 2017. In the last part of this section, the ionospheric parameter of Guangdong Province is analyzed. Section 4 mainly analyzes the impacts of the ionospheric gradient on the GBAS in Guangdong Province. Section 5 presents our conclusions.

## 2. Improved “Simple Truth” Algorithm

The “Simple Truth” algorithm, a precise ionospheric delay calculation algorithm developed by Lee, was used to build the current ionospheric threat model for South Korea [10]. Since a large number of errors were found when using this algorithm to process the GPS data collected in Beijing, an improved “Simple Truth” algorithm was proposed in our previous work [14]. The major difference from the “Simple Truth” algorithm is that the improved “Simple Truth” algorithm implements a more accurate cycle jump detection algorithm and subarc merging algorithm. In this section, first we will describe the basic principles of calculating ionospheric delays and then, we introduce the improved “Simple Truth” algorithm.

**Figure 1:**
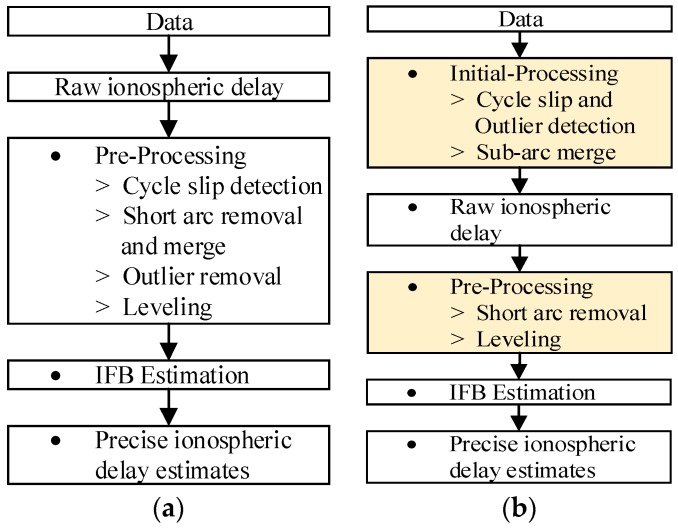
Procedure for the “Simple Truth” algorithm. (**a**) the traditional “Simple Truth” algorithm; (**b**) the improved “Simple Truth” algorithm.

The ionospheric delay can be calculated from dual-frequency (L1 at 1575.45 MHz and L2 at 1227.60 MHz) GPS-range sources. The GPS code ($\rho_{L1}$ $\rho_{L2}$) and carrier-phase ($\phi_{L1}$, $\phi_{L2}$) measurements for the L1 and L2 signal frequencies are as follows [10,15]:

(1) $$\rho_{L1,i}^{k}=r_{i}^{k}+I_{i}^{k}+T_{i}^{k}+\varepsilon_{}$$

(2) $$\phi_{L1,i}^{k}=r_{i}^{k}-I_{i}^{k}+T_{i}^{k}+N_{L1}+\varepsilon$$

(3) $$\rho_{L2,i}^{k}=r_{i}^{k}+\gamma I_{i}^{k}+T_{i}^{k}+c\left(\tau_{i}+\tau_{gd}^{k}\right)+\varepsilon$$

(4) $$\phi_{L2,i}^{k}=r_{i}^{k}-\gamma I_{i}^{k}+T_{i}^{k}+c\left(IFB_{i}+IFB_{}^{k}\right)+N_{L2}+\varepsilon$$

(5) $$\gamma =\frac{f_{L1}^{2}}{f_{L2}^{2}}$$

where $r_{i}^{k}$ is the sum of the geometric ranges between the ith receiver and the kth satellite, receiver clock biases, satellite clock biases and multipath; $I_{i}^{k}$ is the ionospheric delay between the ith receiver and the kth satellite; $T_{i}^{k}$ is the tropospheric delay between the ith receiver and the kth satellite; $N_{Li}$ is the integer ambiguity in the $L_{i}$ (i = 1, 2) frequency carrier phase; $\tau_{i}$ is the receiver inter-frequency bias in the code measurement; $\tau_{gd}^{i}$ is the satellite inter-frequency bias in the code measurement; $IFB_{r}$ is the receiver inter frequency bias in the carrier phase measurement, $IFB^{k}$ is the satellite IFB in the carrier phase measurement; ε is the measurement errors (including multipath errors, antenna errors, etc.) in the code and carrier-phase measurements; and γ represents the ratio of the ionospheric delays I at the L2 frequency to the delay at the L1 frequency. The parameter c is the speed of light in a vacuum. The raw value of the slant ionospheric delay on the L1 frequency can be calculated from the dual-frequency code and carrier-phase measurement in three ways:

(6) $$I_{\rho \_i}^{k}=\frac{\rho_{L2}-\rho_{L1}}{\gamma -1}=I+\frac{c}{\gamma -1}\left(\tau_{i}+\tau_{gd}^{k}\right)+\varepsilon$$

(7) $$I_{\phi \_i}^{k}=\frac{\phi_{L1}-\phi_{L2}}{\gamma -1}=I+\frac{c}{\gamma -1}\left(IFB_{i}+IFB_{}^{k}\right)+\frac{N_{L1}-N_{L2}}{\gamma -1}+\varepsilon$$

(8) $$I_{CMC}=\frac{\rho_{L1}-\phi_{L1}}{2}=I-\frac{N_{L1}}{2}+\varepsilon_{CMC}$$

where the $I_{\rho }$ is dual-frequency code-derived estimate; and $I_{\phi }$ is the dual-frequency carrier-derived estimate. The dual frequency estimates $I_{\phi }$ are prone to semi-codeless tracking errors on L2 measurements, so the code-carrier divergence (CMC) estimates $I_{CMC}$ was computed. $\varepsilon_{CMC}$ is the CMC estimate measurement error. The carrier estimate $I_{\phi }$ has considerably lower multipath and thermal noise errors than those of $I_{\rho }$, but the carrier-phase estimate $I_{\phi }$ contains integer ambiguities for both the L1 and L2 measurements. Therefore, $I_{\phi }$ is used to obtain precise estimates of ionospheric delays and the integer ambiguities $N_{L1}$ and $N_{L2}$ are removed by fitting $I_{\phi }$ to $I_{\rho }$ which combines the advantages of the code and carrier-phase measurements. To compute the finally precise ionospheric delay $I_{DF}$ (dual-frequency ionospheric delay), the receiver and satellite hardware biases must be removed from $I_{\rho }$ and $I_{\phi }$. To perform all these steps, the improved “Simple Truth” algorithm was used, which improved upon the traditional “Simple Truth” algorithm. Figure 1a shows the detailed procedure for the traditional “Simple Truth” algorithm. The input data for the algorithm come in three types, including the GPS RINEX format files containing pseudorange information, navigation files used to compute the satellite position, and satellite IFB data. The output is the accurate ionospheric delay data. The “Simple Truth” algorithm consists of three steps: pre-processing, IFB estimation and precise ionospheric delay estimation [10,16].

The pre-processing includes cycle slip detection, short arc removal and merger, outlier removal, and leveling. Cycle slip detection, performed for each continuous arc, detects cycle slips of carrier-phase observables. Short arc removal and merger remove arcs that have fewer than ten data points or are shorter than five minutes, and merges sub-arcs that are separated by outlier values into continuous arcs. The pre-processing calculates the leveled ionospheric delay, which includes the IFB error. The IFB error is the most severe error in the calculation of ionospheric delay and should be removed. The satellite IFB is available from the Center for Orbit Determination in Europe (CODE), and the receiver IFB can be estimated using the algorithm proposed by Ma and Maruyama [17]. After removing the IFBs of the satellite and the receiver from the leveled ionospheric delay, we can obtain the accurate ionospheric delay $I_{DF}$.

As stated previously, a large number of errors were found when using the traditional “Simple Truth” algorithm to process the GPS data collected from Beijing. We found that the main cause of these errors is that the cycle slip detection and short arc mergers do not work well with poor-quality GPS data. Therefore, we modified the traditional “Simple Truth” algorithm based on the sources of these errors. Figure 1b shows the procedure of the improved “Simple Truth” algorithm. The main improvements are in the algorithm procedure, the cycle slip detections and the sub-arc merge. The traditional preprocessing procedure is divided into the initial-processing and preprocessing steps. The initial-processing step is performed before calculating the raw ionospheric delay. The cycle slip detection and outlier detection are performed simultaneously in the initial-processing step. The modified procedures are indicated in yellow in Figure 1b, and the improvements in the initial processing are as follows:

1. The cycle slip detection and outlier removal are performed on the carrier-phase measurements in the range domain before the initial value of the ionospheric delay is calculated. The method of Melbourne-Wubbena [18] and the ionospheric total electron content rate [19] are simultaneously used to detect the cycle slips and outliers for removal. Because of the influence of noise in the code range observations, the ability of the Melbourne-Wubbena method to detect small cycle slips is limited, although the method can detect large cycle slips well. However, the ionospheric total electron content rate uses only the carrier-phase measurements, so the observational noise is small; therefore, this method can better detect the small cycle jumps. Thus, the combination of the Melbourne-Wubbena method and the ionospheric total electron content rate method can effectively detect cycle slips.
2. The primary function of the subarc merger algorithm is to merge adjacent subarcs, separated into subarcs by outliers, into a continuous arc [10,20]. The traditional algorithm may mistakenly merge subarcs caused by cycle slips rather than by outliers into a continuous arc. The error caused by incorrectly treated cycle slips is then transferred into a leveling error in the incorrect continuous arc. To reduce this error, we propose a dual-fitting method performed on each continuous arc (data gaps are identified as gaps of more than 3600 s between continuous arcs). In this dual-fitting method, a polynomial fit is first performed on the adjacent subarcs $s_{1}\left(t\right)$ and $s_{2}\left(t\right)$ to obtain the polynomials $p_{fit\_s1}\left(t\right)$ and $p_{fit\_s2(t)}$. Second, the residuals $r_{s1}\left(t\right)$ and $r_{s2}\left(t\right)$ are computed at the last point of the first subarc and the first point of the second subarc, as shown in Equations (9) and (10). Third, the difference between $r_{s1}\left(t\right)$ and $r_{s2}\left(t\right)$ is computed using Equation (11). If the largest value of ∇r is less than 0.5 m and the value of $r_{s1}$ is less than 0.8 m, the two adjacent subarcs can be merged into one continuous arc [20].
  (9) $$r_{s1}\left(t\right)=\left|s_{1}\left(t\right)-p_{fit\_s2}\left(t\right)\right|$$
  (10) $$r_{s2}\left(t\right)=\left|s_{2}\left(t\right)-p_{fit\_s1}\left(t\right)\right|$$
  (11) $$\nabla r=\left|r_{s1}\left(t\right)-r_{s2}\left(t\right)\right|$$

This modification of the “Simple Truth” algorithm has three advantages: (1) the cycle slip detection accuracy is increased, (2) the sub-arc merge accuracy is increased by improving the conditions for sub-arc merging, (3) and the outlier removal and cycle slip detection are performed at the same time to simplify the processing without reducing the accuracy. This improved “Simple Truth” algorithm is used to process the GPS data in Section 3 of this paper.

## 3. Statistics and Modeling of Ionospheric Gradient

In this section, we describe the results obtained in Guangdong Province. First, the data sources are introduced, and then the ionospheric gradient statistics are introduced.

### 3.1. Data from the Guangdong GPS Stations

**Figure 2:**
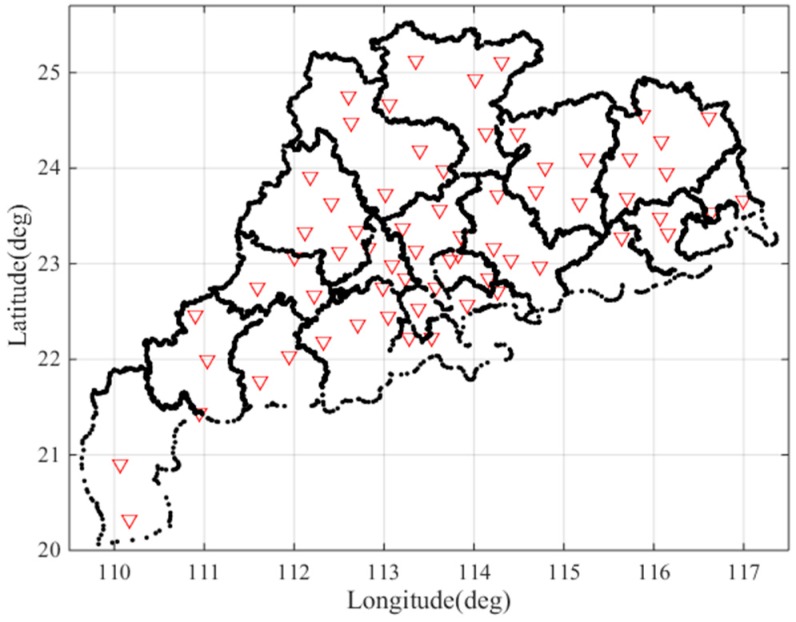
Locations of the Guangdong GPS stations used in this study.

Guangdong Province is a low latitude region located between 20° N and 25° N and between 109° E and 117° E. The GPS data from 21 March 2017, (DOY:080) to 17 April 2017, (DOY:107), were collected at 65 reference stations, as shown in Figure 2, operating in the Guangdong Province. In this figure, a red triangle represents a reference station. The reference stations are evenly distributed and the density of the reference stations is sufficiently high for use in our analysis.

After all the ionospheric delays of the stations were computed, the gradients were computed using the well-known “station-pair method” [13]. The baseline distance between two stations in this work is typically 10–100 km.

### 3.2. Ionospheric Gradient

Guangdong Province is a low latitude region strongly affected by the Sun. The results of this study show that the ionospheric gradient in Guangdong Province is generally larger than that in Beijing [14]. The results revealed two typical anomalous ionospheric events leading to large ionospheric gradients. In the following sections, the two anomalous ionospheric events will be analyzed in detail. The largest ionospheric gradient occurred on 25 March 2017. The second anomalous ionospheric gradient occurred on 24 March 2017. Following the discussions of these two events, we summarize the threat model for Guangdong Province.

#### 3.2.1. Abnormal Condition 1: 25 March 2017

**Figure 3:**
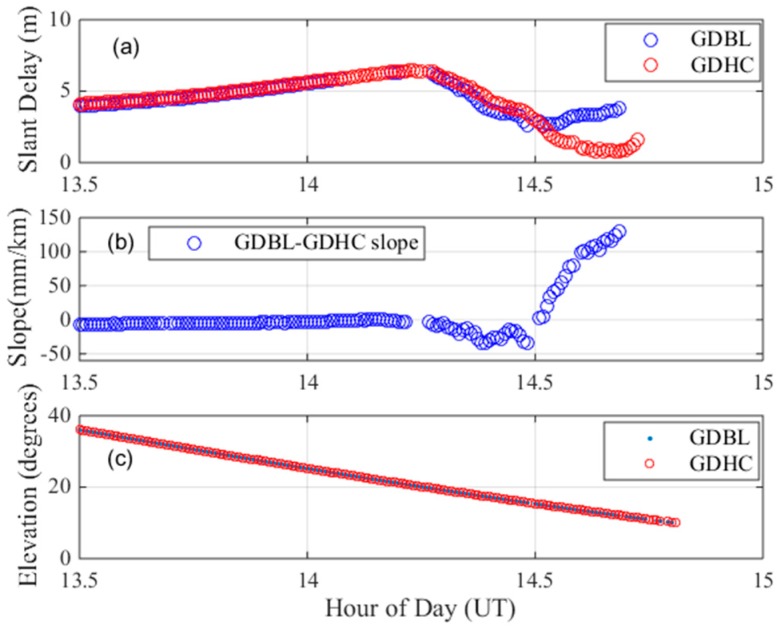
Maximum ionospheric gradient observed on 25 March 2017. (**a**) dual-frequency estimates of slant ionospheric delay; (**b**) ionospheric gradient; (**c**) elevation angles of PRN 30.

On 25 March 2017, a maximum gradient was observed between the GDBL and GDHC stations by the satellite PRN 30. The baseline length between GDBL and GDHC is 23.29 km. Figure 3a shows the dual-frequency delays of PRN 30 over time on this day. The blue line in the figure indicates the ionospheric delay of station GDBL, and the red line indicates the ionospheric delay of station GDHC. No obvious fluctuations in the ionospheric delay occurred before 14.25 h, and the ionospheric delays increased almost the same amount. At 14.25 h, the ionospheric delays of the two stations began to decrease rapidly, and there was a deviation between the delays at the two stations. The ionospheric delay of GDBL was between 4–5 m, and the ionospheric delay of GDHC continued to decrease to 0–2 m. By dividing the differences in the apparent delays by their separation distance, the gradient between these stations is established, as is shown in Figure 3b. The ionospheric gradient between the two stations before 14.25 h was stable at approximately 0 mm/km, although the ionospheric gradient began to fluctuate at approximately 14.25 h; then, the delay increased rapidly, reaching approximately 128 mm/km at approximately 14.75 h. Figure 3c shows the elevation angles over time measured by the GPS satellite PRN 30 from the GDBL (blue) and GDHC (red) stations. Because these stations are close together relative to the viewpoint of a satellite above the Earth’s surface, their elevation angles with PRN30 are almost the same. The largest ionospheric anomaly gradient occurs at the low elevation angle of approximately 18°.

**Figure 4:**
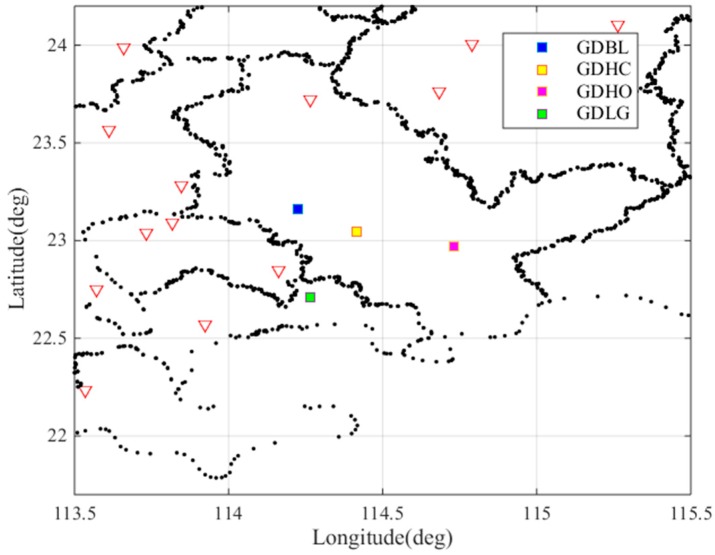
Locations of the nearby stations GDBL, GDHC, GDHO and GDLG.

**Figure 5:**
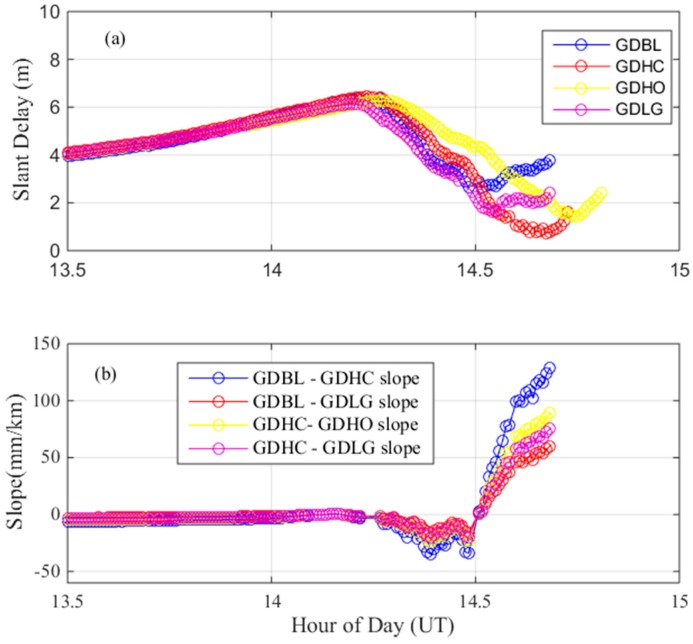
Ionospheric delay and gradient observed from the pairs of nearby stations on 25 March 2017. (**a**) dual-frequency estimates of slant ionospheric delay of nearby stations; (**b**) ionospheric gradient observed form the pairs of nearby stations.

The abnormal event may be caused by receiver fault or false anomalies caused by post-processing error. To verify whether the resulting gradients occurred due to the receiver fault, we studied the nearby pair of stations: GDLG and GDHO. Figure 4 shows the location of these stations. We cross-checked the gradients observed between GDBL-GDLG, GDHC-GDHO and GDHC-GDLG. Figure 5a shows the ionospheric delays of these nearby stations. The purple lines and the yellow lines represent the ionospheric delays of the stations GDLG and GDHO, respectively. Figure 5b shows the ionospheric gradient of the nearby station pairs on 25 March 2017. From the figure we can see that the ionospheric delay and ionospheric gradient in Figure 5 fluctuate between 14.2 h and 14.7 h. It is obvious that the nearby stations were also clearly affected by the abnormal ionospheric activity as opposed to a fault or error on a single receiver fault. Thus, we verified the largest spatial gradients observed between GDBL and GDHC using the ionospheric delays from the nearby stations. Thus, the maximum observed gradient of 128 mm/km is due to an ionospheric event.

**Figure 6:**
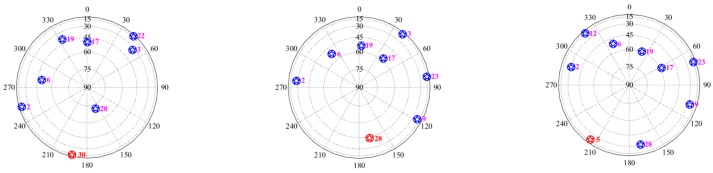
(**left**) Sky plot at 14.75 h on 25 March 2017; (**middle**) Sky plot at 15.5 h on 25 March 2017; (**right**) Sky plot at 15.9 h on 25 March 2017.

To study how many satellites were affected by the abnormal ionospheric events, we calculated the maximum ionospheric gradient for different satellites on 25 March 2017. Table 1 shows the maximum ionospheric anomaly gradients of different satellites from 14 h to 16 h. The PRN 28 satellite is similarly affected by the ionospheric event, with a gradient of 45.5 mm/km; however, the gradient of the PRN 5 satellite is 12.5 mm/km, which is within the normal range. We studied the sky plot map at 14.75 h, 15.5 h and 15.9 h, as shown in Figure 6. We plotted all the satellites at 14.75 h, 15.5 h and 15.9 h. The satellites observed a large ionospheric gradient at each epoch are marked in red. As seen in the figure, when the largest ionospheric gradient occurs, satellite 30, 28 and 5 are at an approximately the 180° azimuth. The affected satellites were also over a remote area. Thus, the ionospheric event affected two satellites (i.e., PRN30 and PRN28).

#### 3.2.2. Abnormal Condition 2: 24 March 2017

**Figure 7:**
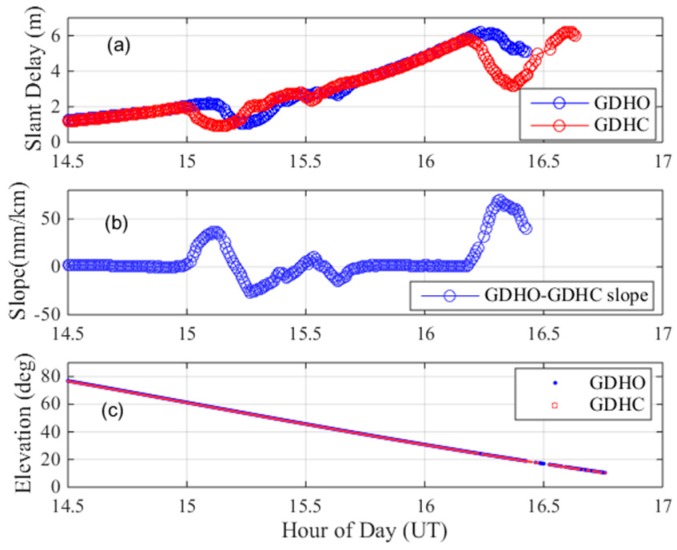
Maximum ionospheric gradient observed on 24 March 2017. (**a**) dual-frequency estimates of slant ionospheric delay; (**b**) ionospheric gradient; (**c**) elevation angles of PRN 28.

The second ionospheric anomaly event occurred on 24 March 2017. We found that there were two ionospheric anomaly events between GDHO and DGHC by viewing the GPS PRN28 on this day, as shown in Figure 7. The distance between GDHO and DGHC is 33.64 km. As shown in Figure 7a, the slant ionospheric delays of the two stations are plotted in red and blue. The first ionospheric event occurred at approximately 15.2 h and the second ionospheric event occurred at approximately 16.3 h. Figure 7b shows the ionospheric gradient between the two stations. The first small ionospheric anomaly event occurred at 15.2 h and resulted in an ionospheric gradient of 44 mm/km. A second more intense set of ionospheric anomalies occurred at approximately 16.3 h and resulted in an ionospheric gradient of 72 mm/km. Figure 7c shows the elevation angles over time of the GPS satellite PRN 28 with GDHO (blue) and GDHC (red).

**Figure 8:**
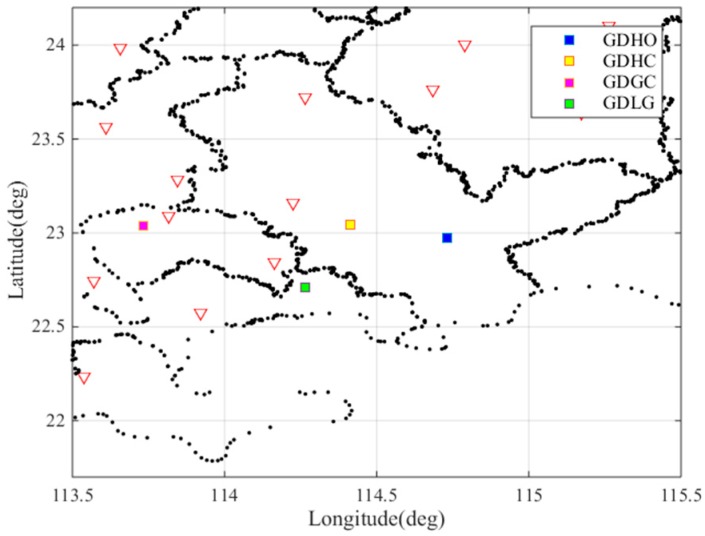
Location of nearby stations of GDHO, GDHC, GDGC and GDLG.

**Figure 9:**
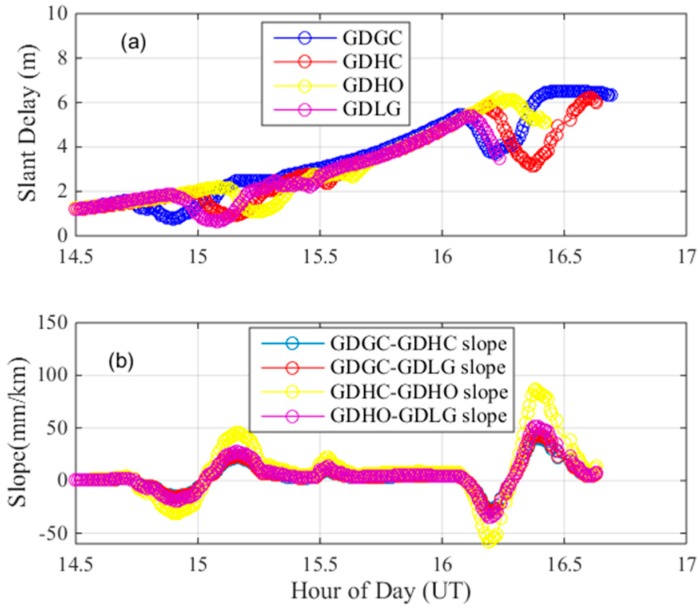
Ionospheric delay and gradient observed from the pairs of nearby stations on 24 March 2017. (**a**) dual-frequency estimates of slant ionospheric delay of nearby stations; (**b**) ionospheric gradient observed form the pairs of nearby stations.

For this abnormal ionospheric event, we also conducted a cross-check to verify its legitimacy. Figure 8 shows the location of nearby stations. Figure 9a shows the estimates of the ionospheric delays of the nearby stations GDGC and GDLG, along with those of GDHO and GDHC. Figure 9b shows the ionospheric gradient of the nearby station pairs GDGC-GDHC, GDGC-GDLG, GDHC-GDHO and GDHO-GDLG on 25 March 2017. As seen in the figure, the nearby stations are also affected by the ionospheric anomaly event, resulting in ionospheric delay fluctuations at approximately 15 h and approximately 16.2 h, meaning that the anomalous ionospheric gradient between GDHO and GDHC is due to an ionospheric anomaly. Two ionospheric events occurred within two hours in one day and should both be given considerable attention.

#### 3.2.3. Ionospheric Threat Model in Guangdong Province

**Figure 10:**
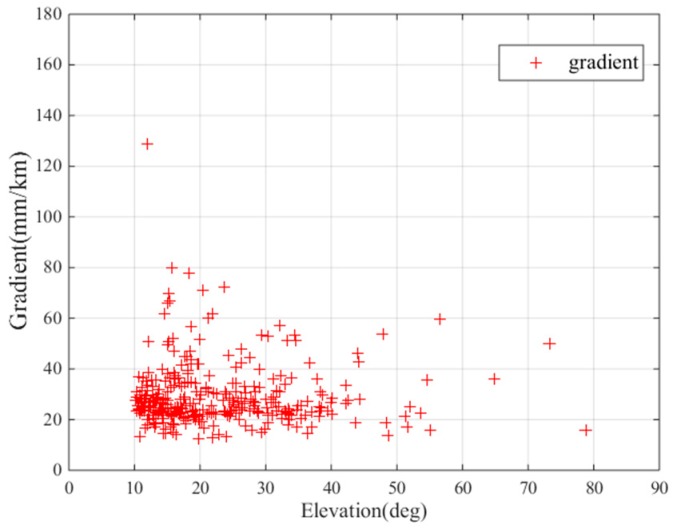
Ionospheric gradient model: ionospheric gradients observed over Guangdong.

Using the improved “Simple Truth” algorithm in Section 2, all GPS data collected at the 65 stations were processed to populate the threat model. We selected all ionospheric anomaly gradients found in Guangdong Province and established a threat model. Figure 10 shows the resulting ionospheric threat model populated with the observations from Guangdong Province. A total of 328 gradients were selected and validated and are plotted along with their corresponding satellite elevation angles. The upper bound of the Korean threat model is 160 mm/km, while the upper bound of the Guangdong gradient is 128 mm/km, which is smaller than that of Korea. The maximum ionospheric gradient observed in Guangdong Province occurred on 25 March 2017. The ionospheric gradient in Guangdong Province is much larger than that in Beijing. Taiwan is at a similar latitude as Guangdong, and thus, its solar radiations are similar, as are its ionospheric activities. The maximum ionospheric gradient found in the Taiwan region is 145 mm/km, which is slightly larger than that of the Guangdong region [21]. When compared to the boundaries of the CONUS, 128 mm/km of Guangdong Province is much smaller, because a much smaller area than CONUS was searched in this paper; additionally, the data we processed did not come from a solar maximum. Therefore, larger anomalous ionospheric gradients may occur in the future and should be continuously monitored with expanded of spatial and temporal scopes.

Similar to the gradient threat models of Korea and Beijing, higher ionospheric gradients were observed at lower elevation in Guangdong [10,14]. This pattern may be related to the limited data, and the spatial characteristics of ionospheric disturbances should be further studied to better determine any satellite geometry dependencies, including satellite azimuths and elevation angles [11].

The standard deviation of the vertical ionospheric gradient $\sigma_{vig}$, which is an integrity parameter broadcast by GBAS, can be used by GBAS users to compute the bounds of the residual ionospheric error [7,12]. Kolb et al. [22] estimated the $\sigma_{vig}$ value as 1 mm/km using local network data in Germany. Lee et al. [13] proposed a conservative $\sigma_{vig}$ value of 4 mm/km to bind the gradients under all ionospheric conditions within the CONUS region. The $\sigma_{vig}$ is also a function of the geographic region. For Guangdong Province, a suitable value needs to be computed.

There are three steps to computing the $\sigma_{vig}$. First, the vertical ionospheric delay is computed. Second, the vertical ionospheric gradient is computed using the station-pair method. Third, the standard deviation ($\sigma_{vig}$) of the vertical ionospheric gradients is were computed. In this study, the slant ionospheric delays are computed using the improved “Simple Truth”. The slant ionospheric delay can be converted into the vertical ionospheric delay using the obliquity factor ($F_{pp}$) in Equation (12) [23]:

(12) $$F_{PP}={\left[1-{\left(\frac{R_{e}\cos\left(\theta \right)}{R_{e}+h_{I}}\right)}^{2}\right]}^{-\frac{1}{2}}$$

where $R_{e}$ is the radius of Earth, $h_{I}$ is the height of the ionospheric shell (approximately 350 km) and θ is the elevation angle of the satellite. The obliquity factor $F_{pp}$ varies from 1.0 with satellites directly overhead to greater than 3.0 with low elevation satellites. To accurately analyze the standard deviations of the vertical ionospheric gradient ($\sigma_{vig}$), this paper selects the data with satellite elevation angles greater than 30° [24].

**Figure 11:**
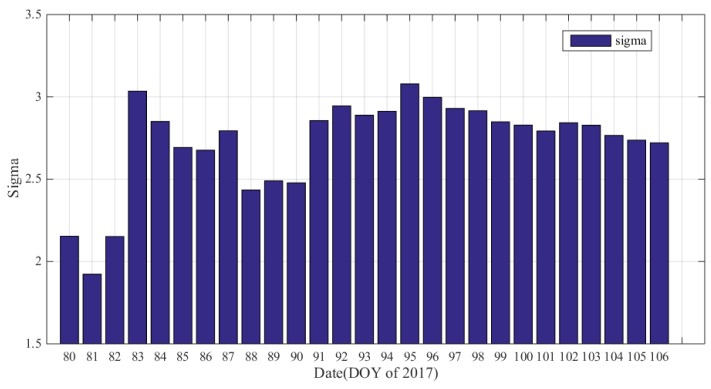
The $\sigma_{vig}$ results on different days.

Figure 11 shows the standard deviations of the vertical ionospheric gradient $\sigma_{vig}$ over the 27 days (DOY: 080-106) studied. As the figure shows, the $\sigma_{vig}$ values is between 2.5 and 3 on most days while only two days have a $\sigma_{vig}$ value exceeding 3 mm/km.

**Figure 12:**
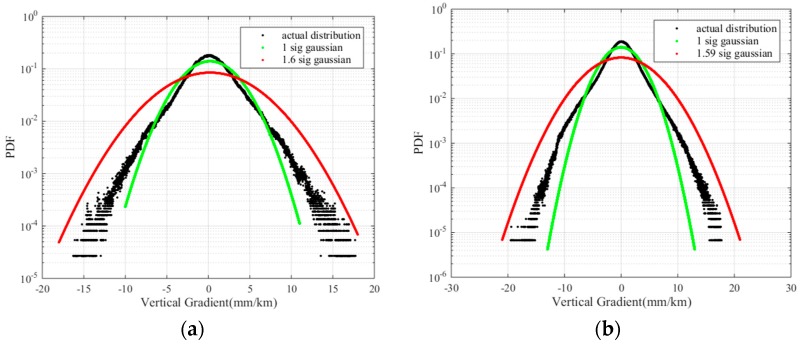
(**a**) Normalized vertical ionospheric gradients on DOY 083; (**b**) Normalized vertical ionospheric gradients on DOY 095.

To fully study the vertical ionospheric gradient $\sigma_{vig}$, we present the probability density function of the vertical ionospheric gradient for the two days (when $\sigma_{vig}$ is greater than 3 mm/km). The distribution of the normalized vertical ionospheric gradients is shown in Figure 12 on a logarithmic scale. The actual distribution shown in the figure has non-Gaussian tails. Because GBAS users assume a zero-mean normally distributed error model in the computation of the protection levels, the nominal sigma (1 σ) of a zero-mean Gaussian distribution(i.e., the green curve shown in the figure) should be inflated to cover the non-Gaussian tails of the actual distribution. We use $\left|\mu_{vig}\right|+f\sigma_{vig}$ to cover the non-Gaussian tails, where $\mu_{vig}$ is the mean of the vertical ionospheric gradients and, f is the inflation factor. As shown in Figure 12 the green curve is a 1 *σ* Gaussian distribution, the black the curve is the actual distribution, and the red curve is the inflated Gaussian distribution. The 1 *σ* values of DOY 083 and DOY 095 are 3.04 mm/km and 3.08 mm/km. The inflation factor of DOY 083 and DOY 095 are 1.53 and 1.51, respectively. The ionospheric gradient (inflated *σ*) was 4.66 mm/km (Figure 12a, DOY:803) and 4.76 mm/km (Figure 12b, DOY:95), respectively. The other days were analyzed, and the ionospheric gradient was less than 4.76 mm/km. Compared with the standard deviation of 4 mm/km in the CONUS, the standard deviation of the vertical ionospheric gradient in Guangdong Province is shown to be larger. To accurately assess the characteristics of the ionosphere in Guangdong, the ionospheric characteristics of the region should be continuously monitored.

## 4. Impacts of Ionospheric Gradients on GBAS

Based on the ionospheric characteristics of Guangdong Province, the effect of the ionospheric gradients on GBAS performances is further investigated in this section. The sigma of the vertical ionospheric gradient is an extremely important parameter in GBAS. Therefore, in this section, the impact of the ionospheric gradient sigma $\sigma_{vig}$ on the VPL of GBAS is first analyzed. Then, we analyze the impact of the maximum anomalous ionospheric gradient on GBAS from an airworthiness perspective.

### 4.1. Simulation Conditions

To evaluate the effects of the ionospheric characteristics on GBAS, the GBAS GAST-D precision approach simulation is performed based on the Guangdong regional ionospheric gradient. The flight speed of the aircraft is 70 m/s, and its starting position is 50 km away from the GBAS (the VHF data coverage is approximately 50 km) [25]. The flight direction is from south to north, and the simulation interval is 0.5 s. The simulation time spans 26 March 2017, 5:15–5:30, and the other variables included in the simulation are summarized in Table 2. It should be noted that the results of the processing may vary with different parameter settings, but they should be universal nonetheless.

From Table 2 $h_{0}$ is the parameter of the tropospheric scale height; $M_{i}$ is the number of ground subsystem reference receivers; GPA is the glide path angle; $v_{air}$ is the speed of the aircraft; $R_{e}$ is the radius of Earth; $h_{I}$ is the parameter of the ionospheric scale height and τ is the time constant of filter.

### 4.2. Impacts of the Ionospheric Gradient Sigma on GBAS

In the case of quiet ionospheric conditions, GBAS can correct the errors caused by ionospheric delays but still contains residual ionospheric errors. Hence, the users need to compute the bounds of the residual ionospheric error with the parameter $\sigma_{vig}$. The VPL is the statistic component used to determine the error bounds in the GBAS, including the ionospheric error, processing error and noise. The GBAS VPL computed for an approach is the maximum of the VPL computed under the *H*0 and *H*1 hypotheses [23]. Here, we consider only the *H*0 case. First, the VPL is given as:

(13) $$VPL_{Apr\_H0}=K_{ffmd}\sqrt{\sum_{i=1}^{N}s_{vert,i}^{2}\;\sigma_{i}^{2}}+D_{V}$$

(14) $$S_{vert,i}=s_{z,i}+s_{x,i}\times \tan\theta_{GPA}$$

(15) $$S={\left(G^{T}WG\right)}^{-1}G^{T}W\equiv \left[\begin{array}{cccc} s_{x,1} & s_{x,2} & \cdots & s_{x,N}\\ s_{y,1} & s_{y,2} & \cdots & s_{y,N}\\ s_{v,1} & s_{v,2} & \cdots & s_{v,N}\\ s_{t,1} & s_{t,2} & \cdots & s_{t,N} \end{array}\right]$$

(16) $$W^{-1}=\left[\begin{array}{cccc} \sigma_{1}^{2} & 0 & \cdots & 0\\ 0 & \sigma_{2}^{2} & \cdots & 0\\ \vdots & \vdots & \ddots & \vdots \\ 0 & 0 & \cdots & \sigma_{N}^{2} \end{array}\right]$$

where $K_{ffmd}$ is a multiplier determined by the probability of a fault-free missed detection, which, in turn, is determined by the integrity risk [26]; i is the ranging source index; $D_{V}$ is a parameter that depends on the active Approach Service Type [23]; N is the number of ranging sources used in the position solution; $s_{vert,i}$ is a projection of the vertical component and the translation of the along track errors in the vertical direction for ith range source (as shown in Equation (14)); and $s_{x,i}$ $s_{z,i}$ are the elements of the S matrix in Equation (15); G is the observation matrix consisting of N rows of line-of-sight vectors from each satellite to the user, augmented by a “1” for the clock; $W^{-1}$ (as seen in Equation (16) is the inverse of the squares weighting matrix (units are in meters squared) [23]; and $\sigma_{i}$ is the pseudorange standard deviation term for the ith ranging source, computed as:

(17) $$\sigma_{i}^{2}=\sigma_{pr\_gnd,i}^{2}+\sigma_{tropo,i}^{2}+\sigma_{air,i}^{2}+\sigma_{iono,i}^{2}$$

where $\sigma_{pr\_gnd,i}$ is the total fault-free noise term provided by the ground function for satellite i; $\sigma_{tropo,i}$ is computed using airborne equipment to derive the residual tropospheric error for satellite i (assumed to be zero in this paper); $\sigma_{air,i}$ is the standard deviation of the aircraft contribution to the corrected pseudorange error for the ith ranging source; and $\sigma_{iono,i}$ is the residual ionospheric delay uncertainty for the ith ranging source and is an important part of $\sigma_{i}$, computed as:

(18) $$\sigma_{iono}=F_{pp}\times \sigma_{vig}\times \left(x_{air}+2\times \tau \times v_{air}\right)$$

where $v_{air}$ is the horizontal velocity of the aircraft (m/s), τ is the time constant of the smoothing filter (s); and $x_{air}$ is the distance between the user and the GBAS station. The VPL calculation process shows that when the vertical ionospheric gradient standard deviation $\sigma_{vig}$ is different, $\sigma_{i}$ and $s_{vert}$ are affected, thereby the VPL is affected.

**Figure 13:**
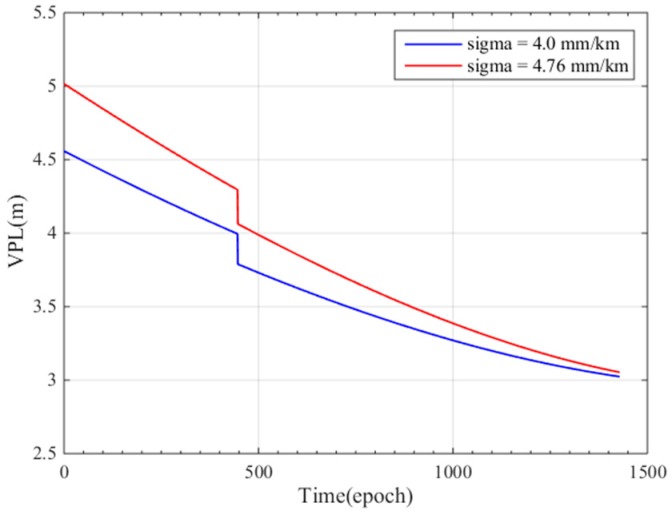
The VPL of a single precision approach under different $\sigma_{vig}$.

To study the influence of different $\sigma_{vig}$ on VPL, we performed a single precision approach landing simulation and computed the VPL using Equation (13). Figure 13 shows the VPL of the single precision approach; the blue line represents the VPL with a sigma of 4.0 mm/km (CONUS), and the red line represents the VPL with a sigma of 4.76 mm/km (Guangdong Province). 

The simulation time interval is 0.5 s and the simulation time is 12 min; A larger $\sigma_{vig}$ indicates that the ionosphere in this region is more active and that the VPL should be greater; therefore, the red curve better represents the positioning error. The figure shows that the impact of changes in sigma decreases as the precision approach progresses; at epoch 0, when the user-GBAS distance is 50 km, the VPL difference is approximately 0.5 m, but when the distance between the user and GBAS is 0, the VPL is almost the same. The difference of the VPL between 4.00 mm/km and 4.76 mm/km is because the different $\sigma_{vig}$ results in different $\sigma_{i}$ in Equation (17). The VPL decreases when the distance between the users and GBAS decreases, as shown in the figure, because the $\sigma_{iono}$ decreases along with $x_{air}$, as seen in Equation (18) At the 447 epoch, VPL changes quickly, because the visible satellite number increases from 10 to 11 and the matrix S (as shown in Equation (15)) changes.

**Figure 14:**
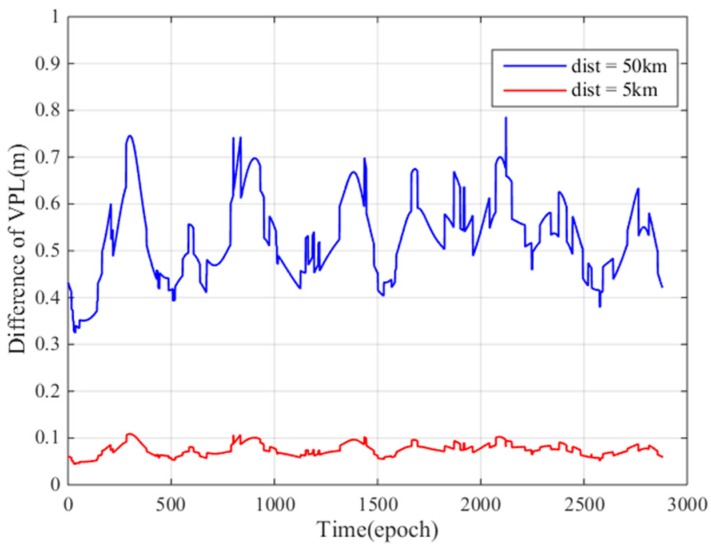
VPL differences when the distances between GBAS are 50 km and 5 km.

To study the maximum VPL difference caused by different $\sigma_{vig}$ values at the GBAS service entrance area and the 5 km ionospheric monitoring point, we fixed users at 50 km and 5 km distances and performed a fixed-point simulation experiment. Figure 14 shows the VPL differences when the distances between the user and GBAS is 50 km and 5 km during a 24 period. The simulation time interval is 30 s. As seen from the figure, at 50 km, the maximum difference between the VPL is 0.8 m (blue curve), and the VPL difference at 5 km is approximately 0.1 m (red curve). During the process of a precise approach, the user should calculate the VPL as accurately as possible; thus, these VPL differences are not negligible, and we should use the $\sigma_{vig}=4.76$ mm/km to compute the VPL.

**Figure 15:**
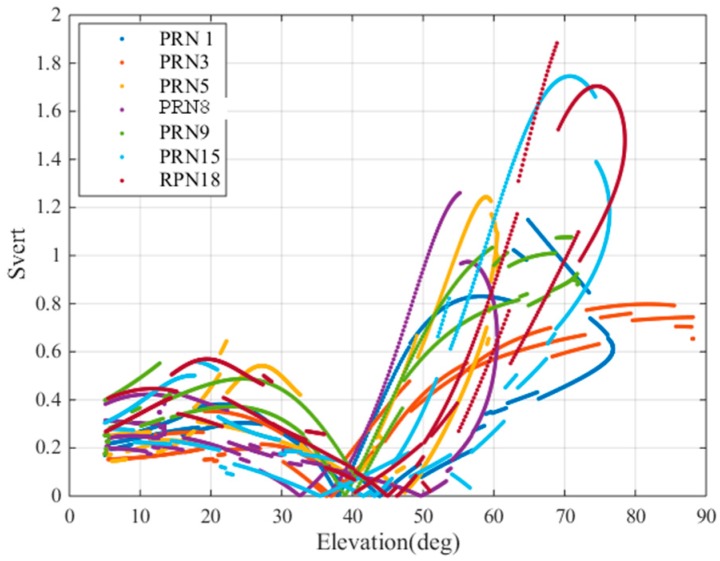
$s_{vert}$ values at different elevation angles.

As mentioned earlier, $s_{vert,i}$ is a projection of the vertical component and the translation of the along track errors in the vertical for the ith ranging source. This parameter has been proposed for use in geometric screenings for GBAS to support CAT III operations [27]. We calculated the $s_{vert}$ values for 24 h based on the geometry and $\sigma_{vig}$ (4.76 mm/km) of Guangdong Province and plotted these values in Figure 15. 

The *x*-axis is the satellite elevation, and the different colors represent different satellites. We selected 7 satellites for the convenience of viewing the figure. As shown in the figure, when the elevation angle is less than 40°, $s_{vert}$ is generally less than 0.8 m. When the elevation angle is greater than 40°, $s_{vert}$ increases as the elevation angle increases, and the maximum $s_{vert}$ is approximately 1.8 for elevations between 70° and 90°. The $s_{vert}$ values of satellites with elevation angles between 30° and 50° are generally lower than those at other elevation angles.

### 4.3. Impacts of Ionospheric Anomaly Gradients on GBAS

This section analyzes the impacts of ionospheric anomaly gradients on GBAS from the perspective of airworthiness. For airworthiness, the user has to have a very high probability of landing within a certain area on the runway (the so-called “touchdown box”) [28,29]. The touchdown box begins 200 ft behind the runway threshold and extends to 2700 ft behind the threshold along the runway for the nominal case (the following discussion is limited to this case). The touchdown requirements can be converted to vertical requirements and thus, converted to a pseudo range domain, i.e., the difference correction error should be within the pseudorange error limits. We compare the maximum difference correction error and the difference correction limits. When the difference correction error is greater than the limit, it will cause airworthiness risk.

To derive the differential correction error limit between a GBAS and user, we show the origin of the airworthiness limits. The touchdown requirements are related to the total system error (TSE) of an aircraft, and the TSE contains the navigation system error (NSE) and the flight technical error (FTE). Descriptions of how the allocation of the two contributors and the mapping form the touchdown requirements into an error bound for an individual pseudorange can be found in [30,31]. The critical component of the TSE error is its vertical component, which is called $E_{v}$; it contains the nominal error component and the ionospheric anomaly error $E_{v,iono}$. Taking the nominal NSE and FTE as the Gaussian distributed random variables, $E_{v,iono}$ conforms to the following formula [30]:

(19) $$200ft\leq NTDP-\frac{NSE_{vert,ff,95\%}+E_{v,iono}}{\tan(GPA)}-FTE_{ff,95\%}$$

where NTDP denotes the nominal touchdown point on the runway, which is generally assumed to be located 1290 ft behind the runway threshold. When the GPA is 3°, the maximum vertical error limit would be $E_{v,iono}\leq 8.4\;m$. This error limit in the position domain can be converted into the pseudorange domain based on the relation between the pseudorange domain and the position domain as given as $s_{vert}$ in Equation (14):

(20) $$E_{r,i,\max}=\frac{E_{v,iono}}{S_{vert,i}}$$

**Figure 16:**
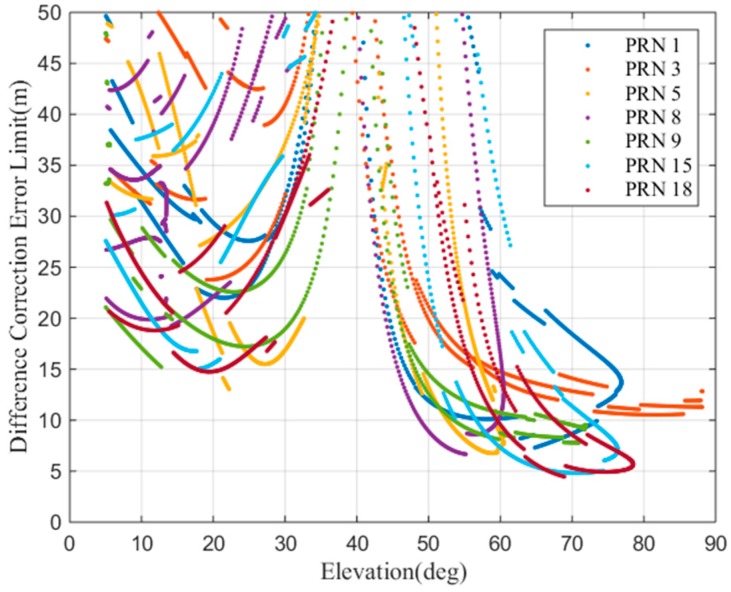
The actual calculated difference correction error limit.

In [30], $s_{vert}$ is assumed to be 4, which means that the contribution of a single satellite to a vertical position error with respect to the approach track cannot be larger than four times. As shown in Figure 15, $s_{vert}$ is a function of the satellite elevation and is smaller than 4. Thus, in this work, we use the actual calculated value. The $E_{r,i,\max}$ is the actual differential error limit. Figure 16 is the computed difference correction error limit as a function of the satellite elevation. From the figure, we can see that, when the satellite elevation is approximately 20° and above 50°, the difference correction limit is relatively small. When the elevation of the satellite is above 50°, the differential error limit is lower. This shows that a high elevation satellite is more likely to suffer airworthiness risks.

We compare the computed $E_{r,i,\max}$ with the actual differential error to see whether it can meet the airworthiness requirements. To calculate the maximum differential error, we simulated the ground subsystem and the user subsystem of the GBAS according to the actual satellite geometry and ionospheric parameters of Guangdong. By simulating the ionosphere wave fronts at both the aircraft and GBAS levels, we find the resulting maximum differential errors. To facilitate the study, we first assume the parameter of the ionospheric gradient, as shown in Table 3.

**Figure 17:**
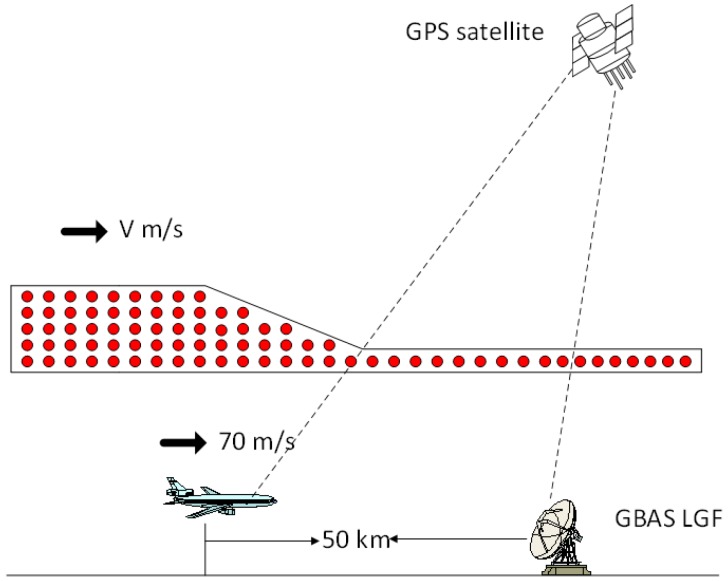
One of the worst-case scenarios.

To find the greatest impact of the ionospheric anomaly gradient, we simulate the worst-case scenario, i.e., when the ionospheric anomaly comes from behind and catches up with the user before passing them, as shown in Figure 17. 

When the user or the GBAS signal is affected by the ionosphere, the true pseudorange is added to the real ionospheric delay, and then, Hatch filtering is performed at the user and ground sides. The difference correction is calculated at the ground for the user [32,33]. If both the user and GBAS see the same ionospheric delay, then no impact is seen because the user error induced by the ionosphere is cancelled out when the differential corrections broadcast by the GBAS are applied. In the worst-case scenario, there is a window where no ionospheric correction is available to the user.

**Figure 18:**
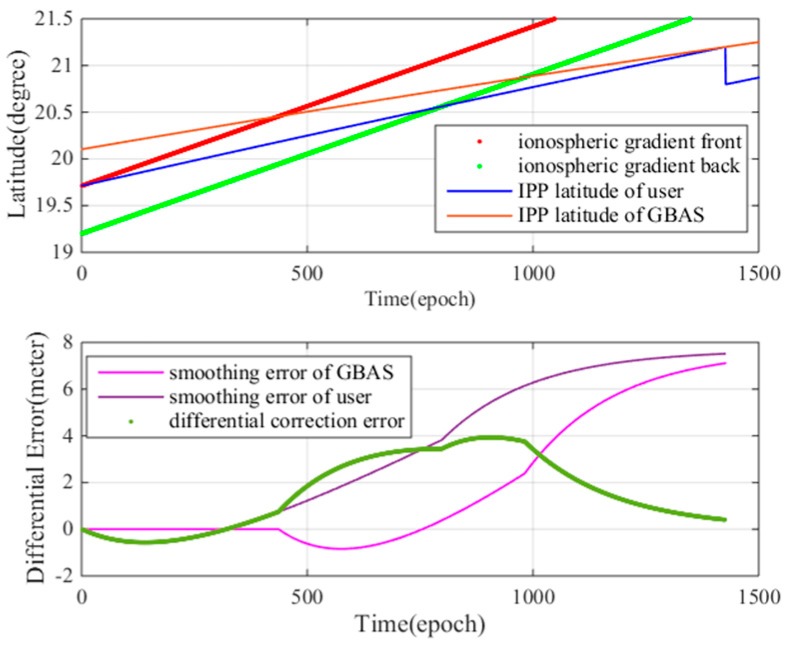
(**a**) relative position between the abnormal ionospheric gradient and the GBAS IPP; (**b**) ionosphere gradient impact on GBAS.

Figure 18 shows the impact of the ionospheric gradient of PRN 13 on the user and GBAS. The x-axis is the time of the user approach, with a time interval of 0.5 s. Figure 18 (top) shows the relative position between the abnormal ionospheric gradient and the GBAS IPP (Ionospheric Penetration Point) and the user IPP. As shown in Table 3, the ionospheric velocity is 400 m/s, while the aircraft velocity is 70 m/s. The ionospheric gradient reaches the user IPP and continues onward. As in the figure, the red and green lines represent the latitudes of the front and back of the ionospheric gradient, respectively. When the IPP of one satellite is located between the red line and green line, the satellite is affected by the ionospheric gradient.

In Figure 18 (bottom), the *y*-axis is the ionosphere-induced error between the GBAS and the user. The green line represents the differential correction error after the differential correction. As shown in the figure, the differential correction error changes considerably in response to each state of the top figure. At epoch 0 and 475, the resulting error is negative because of the ionospheric delays within the pseudorange and advances of the carrier-phase measurements; when the user starts to see the gradient, the impact on the carrier phase dominates. When the front of the ionosphere gradient begins to affect the GBAS (at epoch 475), the differential correction errors increases rapidly and reaches a maximum value of 4 m at approximately epoch 900 because GBAS cannot provide differential corrections when the ionospheric gradient affects GBAS.

**Figure 19:**
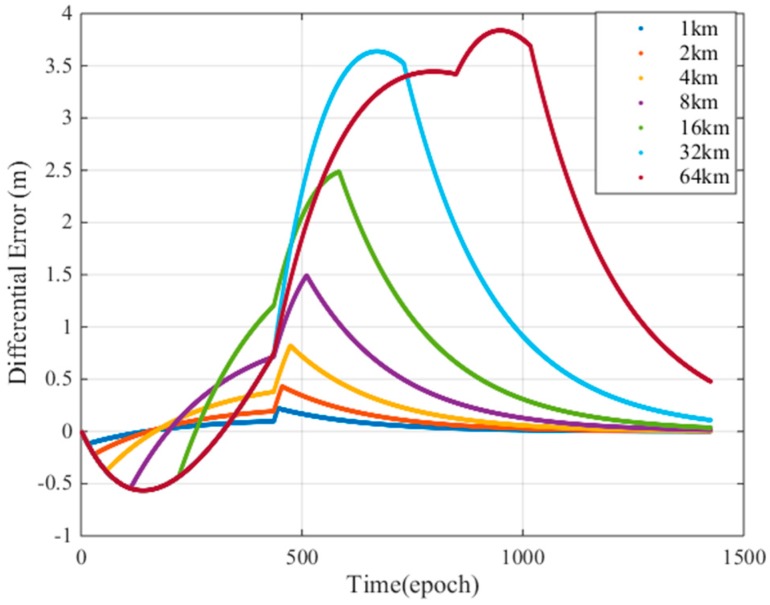
Differential error due to ionospheric gradient thickness.

**Figure 20:**
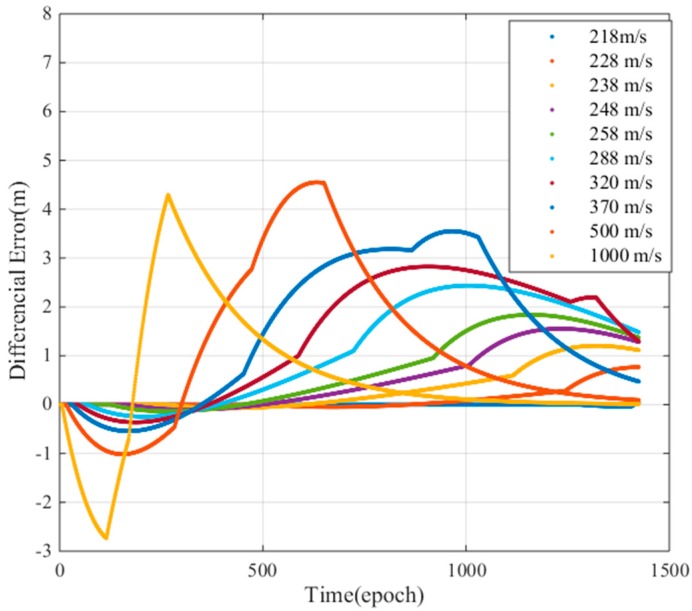
Differential error due to ionospheric gradient speed.

To fully study the influence of the ionospheric gradient on GBAS, this work analyzes the influence of different ionospheric velocities and the widths on the differential correction errors for the 128 mm/km gradient. Figure 19 shows that the width of the ionospheric gradient has an impact on the differential error. The differential correction errors of the GBAS change for different ionospheric widths. We must find an ionospheric width that makes the difference correction error reach its maximum value. Figure 20 shows that the speed of the ionospheric gradient has a similar impact on the differential error. As seen in the figure, the different speeds and widths of the ionospheric gradient lead to differential correction errors; thus, we compute the largest differential correction error by changing the velocity and width of the ionospheric gradient. As Figure 16 showed earlier, the differential error limit is related to the satellite elevation. Therefore, we analyze the two cases of low elevation angles and a high elevation angles. Based on our simulations, at low elevation conditions, satellite 13 is affected by the ionospheric gradient and at high elevation conditions, satellite 18 is affected by the ionospheric gradient. For these two cases, the ionospheric gradient velocities and widths must be computed to determine the maximum differential correction error.

**Figure 21:**
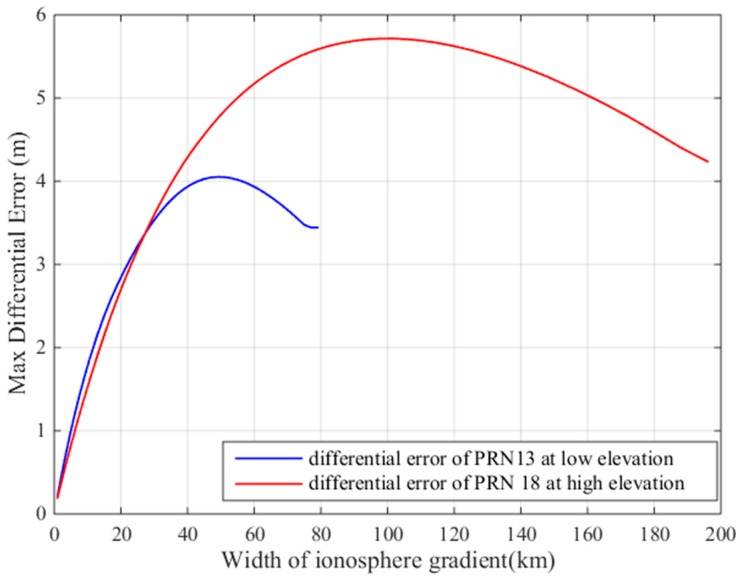
Differential error vs. ionospheric gradient width.

**Figure 22:**
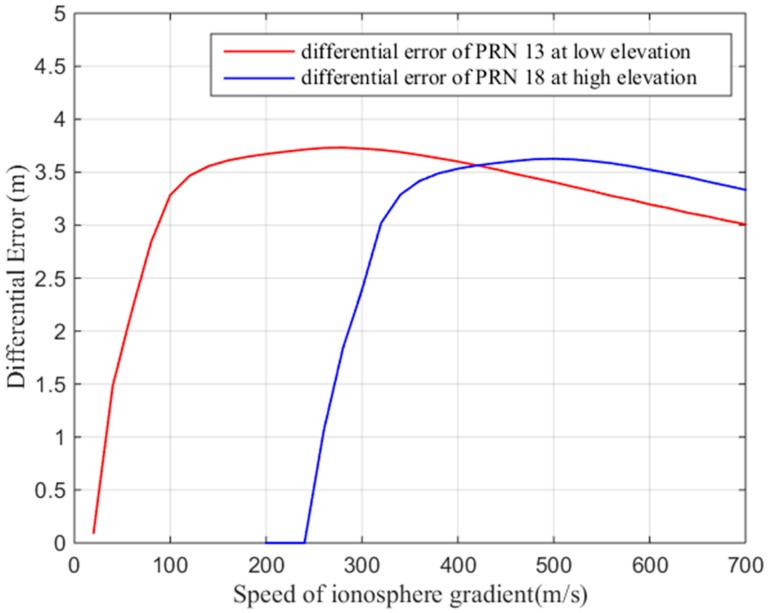
Maximum differential error vs. ionospheric gradient speed.

Figure 21 shows the resulting differential error as a function of the ionospheric gradient width. As shown in the figure, when the gradient width is 50 km, the difference correction error of PRN 13 is the largest, and when the gradient width is 100 km, the difference correction of PRN 18 is the largest. Figure 22 shows the resulting maximum differential error as a function of the ionosphere gradient speed. When the gradient speed is 500 m/s, the difference correction error of PRN13 is the largest and when the gradient speed is 280 m/s, the difference correction error of PRN18 is largest. Therefore, for low elevations, we choose a maximum speed of 500 m/s and a width of 50 km to compute the maximum ionospheric difference correction error, and for high elevations, we choose a maximum speed of 280 m/s and a width of 100 km to compute the maximum ionospheric difference correction error.

**Figure 23:**
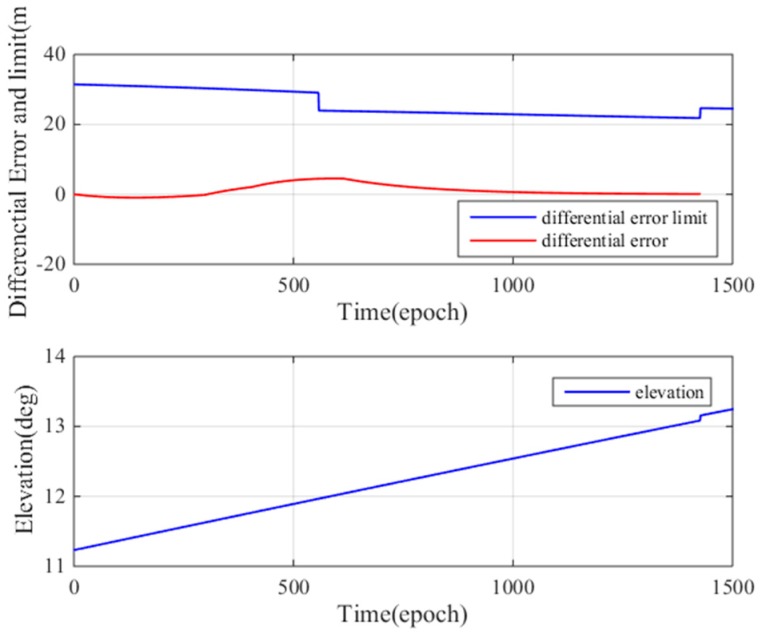
(**top**) Maximum differential error versus ionospheric gradient width at high satellite elevation; (**bottom**) satellite elevation.

**Figure 24:**
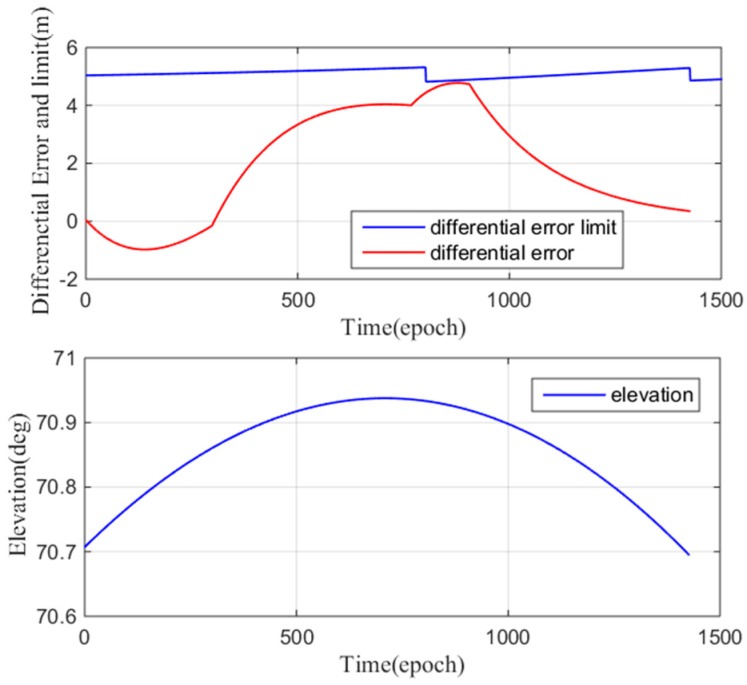
(**top**) Maximum difference correction error and the monitoring threshold at high satellite elevation; (**bottom**) satellite elevation.

Figure 23 (top) shows the low elevation results. In the precision approach simulation, the differential error limit (computed using Equation (20)) is approximately 30 m, and the maximum difference correction error is 5 m. Figure 23 (bottom) shows the satellite elevation (11–13°). In this case, the maximum differential correction error does not exceed the differential correction error limit, the user will land safely at the designated landing position, and thus, the maximum ionospheric gradient meets the airworthiness requirements. Figure 24 shows the high elevation results. The difference correction error limit and the difference correction error are calculated in real time. Figure 24a shows that at epoch 900, the differential error limit is approximately 5 m, and the maximum difference correction error is slightly lower than 5 m. In this case, the maximum differential correction error is almost equal to the differential correction error limit, which means that the current ionospheric gradient may cause the user to land outside the correct landing area. In this situation, the user may fail to land. The maximum ionospheric gradient cannot meet the airworthiness requirements.

We analyzed the airworthiness of the maximum differential correction errors at low and high elevations. The maximum differential correction errors in both cases are approximately 5 m; the maximum differential correction error can meet the airworthiness requirements for low elevation conditions but may not be able to meet the airworthiness requirements for high elevation conditions. The results of this analysis are universal: when a satellite cannot meet the airworthiness requirement, GBAS must remove the satellite, or even refuse its service.

## 5. Conclusions

Using the improved “Simple Truth” algorithm, the data collected from 65 GPS stations in Guangdong Province were processed. We observed and validated the spatial gradients in the slant domain that were as high as 128 mm/km and 72 mm/km during two different ionospheric anomalies. The vertical ionospheric gradient sigma $\sigma_{vig}$ is 4.76 mm/km. A total of 328 ionospheric gradients were selected. The gradients were plotted with their corresponding satellite elevation angles, and an ionospheric gradient model was established.

The influence of the ionospheric gradient on the GBAS in Guangzhou Province has been evaluated. We found that when the user begins to enter the GBAS service area, the vertical ionospheric gradient sigma value of 4.76 mm/km in the Guangdong region increases the VPL value by 0.8 m. Additionally, the $s_{vert}$ values of the satellites with elevation angles between 30° and 50° are generally lower than those at other elevation angles, which shows that the high elevation satellites are more easily affected by ionospheric anomalies. We analyzed the differential correction errors caused by ionospheric anomalies at low and high elevation angles. When a satellite is at low elevation, the $s_{vert}$ is smaller and the difference correction limit is larger. The differential correction error caused by ionospheric anomalies does not lead to an airworthiness risks for these low elevation satellites, but for high elevation satellites, the $s_{vert}$ is relatively large and may be as high as 2; thus, the difference correction limit is smaller. In the case of the Guangdong maximum ionospheric gradient, the maximum differential correction error of GBAS may be 5 m, which can cause an airworthiness risk for the high elevation situations. This discovery provides a reference for the GBAS satellite selection method; when ionospheric storms occur, the use of extremely high elevation satellites should be reduced.

## Figures and Tables

**Table 1:** Maximum ionospheric anomaly gradients for different satellites.

PRN	Time	Gradient
30	14.75 h	128 mm/km
28	15.5 h	45 mm/km
5	15.9 h	12.5 mm/km

**Table 2:** Parameters used in the simulation.

Parameter	Description
Ground Accuracy Designator	GAD-C
Aircraft Accuracy Designator	AAD-B
$h_{0}$	7.6 km
$M_{i}$	4
GPA	3°
$v_{air}$	70 m/s
$\sigma_{vig}$	4.76 mm/km
$R_{e}$	6378.1363 km
$h_{I}$	350 km
τ	100 s
Max ionospheric gradient	128 mm/km

**Table 3:** Ionospheric anomaly simulation parameters.

Parameter	Description
Ionospheric velocity	400 m/s
Ionospheric direction	South to north
Ionospheric tip width	60 km
Ionospheric gradient	128 mm/km
